# Motor Imagery-Based Brain-Computer Interface Combined with Multimodal Feedback to Promote Upper Limb Motor Function after Stroke: A Preliminary Study

**DOI:** 10.1155/2021/1116126

**Published:** 2021-11-03

**Authors:** Yi-Qian Hu, Tian-Hao Gao, Jie Li, Jia-Chao Tao, Yu-Long Bai, Rong-Rong Lu

**Affiliations:** ^1^Department of Rehabilitation, Huashan Hospital, Fudan University, No. 12 Middle Wulumuqi Road, Shanghai 200040, China; ^2^Department of Computer Science and Technology, Tongji University, No. 4800 Cao'an Highway, Shanghai 200092, China

## Abstract

**Background:**

Recently, the brain-computer interface (BCI) has seen rapid development, which may promote the recovery of motor function in chronic stroke patients.

**Methods:**

Twelve stroke patients with severe upper limb and hand motor impairment were enrolled and randomly assigned into two groups: motor imagery (MI)-based BCI training with multimodal feedback (BCI group, *n* = 7) and classical motor imagery training (control group, *n* = 5). Motor function and electrophysiology were evaluated before and after the intervention. The Fugl-Meyer assessment-upper extremity (FMA-UE) is the primary outcome measure. Secondary outcome measures include an increase in wrist active extension or surface electromyography (the amplitude and cocontraction of extensor carpi radialis during movement), the action research arm test (ARAT), the motor status scale (MSS), and Barthel index (BI). Time-frequency analysis and power spectral analysis were used to reflect the electroencephalogram (EEG) change before and after the intervention.

**Results:**

Compared with the baseline, the FMA-UE score increased significantly in the BCI group (*p* = 0.006). MSS scores improved significantly in both groups, while ARAT did not improve significantly. In addition, before the intervention, all patients could not actively extend their wrists or just had muscle contractions. After the intervention, four patients regained the ability to extend their paretic wrists (two in each group). The amplitude and area under the curve of extensor carpi radialis improved to some extent, but there was no statistical significance between the groups.

**Conclusion:**

MI-based BCI combined with sensory and visual feedback might improve severe upper limb and hand impairment in chronic stroke patients, showing the potential for application in rehabilitation medicine.

## 1. Introduction

Stroke remains the leading cause of long-term disability, among which upper limb paralysis is particularly common and prevents patients from returning to self-care [1]. Rehabilitation has been shown to recover some motor functions from stroke. Several clinical controlled trials have confirmed that it is not hard to regain partial control of the shoulder and elbow from positive and intensive rehabilitation training [2–4]. However, it is difficult to improve the recovery of the control of severely paretic wrist and fingers, which prevents patients from returning to family and society [5]. Hence, there exists a pressing need to seek new interventions to improve the motor function of the paralysed upper limb. Sensory disorders, including deep and superficial sensory, are also common in stroke patients [6–8]. Studies have shown that the recovery of sensory impairments promotes the recovery of motor impairments; hence, increasing sensory input and output during exercise training may play an important role [9–11].

The brain-computer interface (BCI) is new technology that has been developing rapidly, and it directly acts on the central nervous system. By collecting and analysing the biological electrical signals from the human brain, BCI technology could establish a direct communication and control pathway between the human brain and computers or other electronic devices [12]. This unconventional pathway has the potential to help stroke patients regain movement [13–15]. There are many ways to generate brain signals, among which motor imagery (MI) is more common [16,17]. The phenomenon of event-related desynchronisation (ERD)/event-related synchronisation (ERS) occurs when patients perform motor imagination [18]. In fact, many rehabilitation interventions are not appropriate for severely paralysed stroke patients, which means that these patients are excluded from therapy [19]. MI requires patients to have good cognitive function and can be carried out after standardised training and guidance, even for patients with severe hemiplegia [20–22]. Previous clinical studies have shown that MI or a combination of it and physical therapy can promote recovery of the upper extremities, which is important for daily activities and skills [23–25]. EEG is widely used because of its noninvasive, portability, and good temporal resolution. Researchers have demonstrated that MI-based BCI has the potential to be used in stroke rehabilitation [26–28].

BCI training is often combined with virtual reality (VR), which provides a three-dimensional visual interaction during training [29]. Adding VR to BCI might arouse patients' interest, which is conducive to improving patient compliance [30]. Adverse reactions such as dizziness and eyestrain might occur during treatment.

In previous research, BCI was mostly used as an intermediate medium to output signals that can control external devices, such as exoskeleton robots and functional electrical stimulation (FES) [31,32]. Studies have confirmed its clinical efficacy, and the improvement has a certain degree of continuity [33]. In our previous study [34], continuous passive motion (CPM) was used as an external device to improve wrist extension in chronic stroke patients. The results showed that after six weeks of interventions, 81% of the patients who completed the study had regained wrist extension. Meanwhile, the spatial and spectrum pattern of the EEG signal also improved after the training. However, this was before and after study and did not set up a control group. Also, external devices might have some insufficiencies, such as economic costs and patient adaptability. Patients might mainly focus on internal motor imagery during MI-based BCI training if external devices do not exist. In the current study, we further investigated the effect of sensory, visual, and EEG feedback on the recovery of upper limb motor function in chronic stroke patients.

## 2. Methods

### 2.1. Participants and Study Design

Twelve stroke patients were enrolled in this preliminary study. The inclusion criteria for participation were as follows:

1. Diagnosis as cerebrovascular diseases confirmed by CT or MRI;
2. No active extension of the paretic wrist and manual muscle test (MMT) for wrist extension at grade 0–1;
3. Disease duration more than two months;
4. Without cognition impairment (Mini Mental State Examination (MMSE) score >21); and
5. No hearing or visual impairment.

The exclusion criteria included the following:

1. The kinaesthetic and visual imagery questionnaire (KVIQ) [35] showing the MI task could not be performed, with a score less than 25;
2. Patients with contraindications for MRI; and
3. Participation in other clinical trials.

The current study is a single-centre, single-blinded prospectively randomised controlled study. The intervention was given by a qualified physiatrist, and another physiatrist who did not know the group of participants assessed all the participants at baseline and immediately after all the interventions. The participants were randomly divided into two groups: the MI-based BCI group and the classic MI group. All of them received individualised rehabilitation therapy based on their impairment. The scheme was carried out in accordance with the Helsinki Declaration and approved by the local ethics committee of Huashan Hospital. All the participants signed a written informed consent form before treatment. The clinical trial was registered (ChiCTR2000034725).

### 2.2. EEG Signal Acquisition and Processing

Only participants in the BCI group would get their EEG signals collected and processed. An 11-channel high-resolution EEG system (g.USBamp, g.tec, Schiedlberg, Austria) was used, and electrodes were attached to the scalp according to the 10–20 international system standard as follows: “FC3,” “FC4,” “C5,” “C3,” ”C1,” “CZ,” “C2,””C4,” “C6,” ”CP3,” and “CP4”. The ground electrodes were placed on the medial frontal cortex. The reference electrodes were, respectively, fixed at the left and right mastoids, and the average value from bilateral electrodes was used as the reference. EEG signals were collected at a sampling rate of 256 Hz.

Then, we followed the instructions to preprocess raw EEG data, as suggested by Makoto Miyakoshi's recommendation [36]. The pipeline is as follows:

1. Import EEG data;
2. High-pass filter the data at 8 Hz and low-pass filter the data at 30 Hz;
3. Import channel info and use Montreal Neurological Institute (MNI) coordinate file for boundary element model (BEM) dipfit model;
4. Remove line noise using CleanLine;
5. Apply clean_rawdata to reject bad channels and correct continuous data using artefact subspace reconstruction (ASR);
6. Interpolate all the removed channels;
7. Reference the data to average; and
8. Epoch data from −1 to 4 sec to event onset.

Power spectrum density (PSD) and individual channel's event-related spectral perturbation (ERSP) were used to analyze the MI-related activities.

### 2.3. Classic Motor Imagery Training

The classic MI group was set as the control group. The participants in this group also received 30 minutes of motor imagery training (MIT) per day. MIT was given in a fixed quiet room, and the training implementation was divided into four steps. One qualified therapist conducted all the training. The patients closed their eyes and listened to the therapist's instructions to complete the corresponding imagination tasks. The MIT procedure was as follows: first, patients were instructed to relax their body for two to three minutes. Then, they were told to close their eyes and imagine lying in a warm, relaxing place (such as a sofa, beach, or lawn). Second, the patients were prompted to perform MI of each joint in the upper limb for 10 minutes, including flexion and extension of the shoulder, elbow, wrist and fingers, and pronation and supination of the affected forearm. Third, for the next 15 minutes, patients were asked to imagine familiar, daily hand movements with their paretic upper limb, for example, “extending the affected arm to touch the red apple in front and then withdrawing the arm back” or “reaching out and grasping a cup on the table, bringing it to the mouth and reaching out to put it back.” The content of the imagery task could be combined with occupational therapy, and the therapist could adjust the content of each MI task. Finally, the therapist directed the patient to return their attention to their surroundings within two to three minutes. To inform the patients that they were back in the room, the therapist asked them to listen to the sounds around as the therapist slowly counted down from 10 to 1. Then, the patients slowly opened their eyes while the therapist counted to one [37, 38]. To monitor whether the patients were cooperating with the imagery, the therapist could ask the patients about the fidelity and clarity of the imagery movements during the process to better help them enter the imagery state.

### 2.4. MI-Based BCI Training

Before intervention, the patients were first fitted with an EEG cap and plugged into the electrode with a conductive paste. During the intervention, the patients received both sensory input (with a brush) in both hands and visual feedback (the movements of virtual hands with VR glasses). The procedure of MI-based BCI training is as given below.

**Figure 1:**
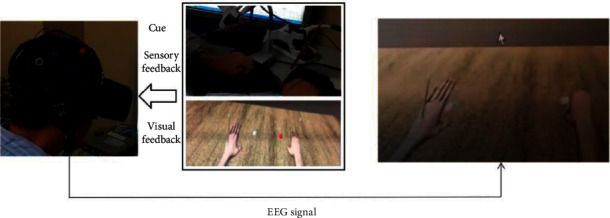
Illustration of the MI-BCI intervention procedures combined with multimodal feedback.

The patients first received sensory feedback in the back of both hands. After the sensory input stopped, grasping or extending actions of right/left virtual hand appeared randomly. After seeing the action of the virtual hand in the screen, the patients needed to perform the MI task of the same side simultaneously, namely, by grasping or extending this certain hand. The real-time EEG signals were collected and analysed by a computer to extract the characteristics of EEG patterns. If this pattern was in accordance with the preset pattern, the virtual hand would display the movements (grasping or extending) according to the MI of the patients. So, the patient received both sensory and visual feedback during the process. The above process was repeated during the intervention. Each session lasted 30 minutes (four cycles of six minutes each with two-minute intervals). The whole procedure is listed in Figure 1.

### 2.5. Clinical Outcome Measures

The primary outcome measure was the motor part of the Fugl-Meyer assessment-upper extremity (FMA-UE) score, which is widely recommended for the evaluation of motor impairments in stroke rehabilitation research.

Secondary outcome measures included the following: (1) action research arm test (ARAT); (2) motor status scale (MSS); (3) increased range of motion (ROM) of the affected wrist or the amplitude and cocontraction of the extensor carpi radialis on the surface electromyography (sEMG); and (4) Barthel index (BI).

### 2.6. Statistical Analysis

For clinical outcome measures, SPSS (version 20.0, Chicago, IL, USA) was used for all analyses. The general *χ*2 test or Fisher's exact test was used in the enumeration data. Paired sample *t*-tests were used to compare the differences within the groups, and an independent sample *t*-test was used to determine the difference between groups. The parameters of the major efficacy evaluation indicators were estimated, and the 95% confidence intervals (CI) of the differences before and after the intervention were given. Significant differences were considered if *p* < 0.05.

For EEG data, EEGLAB was used to perform all preprocessing routines and statistical analyses. For each participant, we performed a simple two-tailed paired *t*-test at each trial. We used 0.05 for the bootstrap significance level. For PSD, *p* values were computed at every frequency; for ERSP, *p* values were computed at every time/frequency point. Only those results with *p* < 0.05 showing statistical significance were preserved.

## 3. Results

### 3.1. Participants

Twelve stroke patients were enrolled and randomly divided into two groups: seven in the MI-BCI group and five in the MI group. All patients completed the entire training and assessment sessions. The demographic characteristics are listed in Table 1.

## 4. Clinical Outcome Analysis

### 4.1. Primary Outcome Measure

Compared with baseline, the FMA-UE score of the experimental group improved significantly (*p* = 0.006), while the control group did not show a significant improvement (*p* = 0.068). However, there was no significant difference when comparing the two groups (Table 2).

### 4.2. Secondary Outcome Measures

After treatment, both groups showed significant differences in MSS scores. The score for MI-based BCI increased from 19.17 ± 10.33 to 15.94 ± 8.97 (*p* = 0.010), while the score for classic MI was from 23.90 ± 11.11 to 18.60 ± 10.98 (*p* = 0.002).

The ARAT scores and modified BI did not differ significantly between the two groups (*p* = 0.375 and *p* = 0.376, respectively). After MI-based BCI training, the scores of the ARAT and the BI were 5.57 ± 7.66 and 69.00 ± 11.93, respectively, while after classic MI training, the scores of the ARAT and the BI were 8.60 ± 9.10 and 72.00 ± 6.71, respectively.

All the participants were unable to actively extend their affected wrists at baseline. After completing all the training, two patients from each group were able to actively extend the paretic wrist. Further analysis of the surface electromyogram (sEMG) results showed a certain degree of improvement in each group, but no significant difference was observed within or between the two groups in the amplitude change of the extensor carpi radialis longus (*p* = 0.305 and *p* = 0.130, respectively) (Table 2).

## 5. Analysis of EEG Signals

### 5.1. Frequency Analysis

For the time-frequency analysis of EEG, we used ERSP as an indicator. According to prior knowledge, when a healthy person performs a left or right hand motor imagery task, oscillations in the alpha and beta bands can display either an event-related blocking response or an event-related amplitude enhancement. The former is named event-related desynchronisation (ERD) and the latter event-related synchronisation (ERS). In ERSP measurements, ERD indicated a power spectrum reduction, while ERS indicated a power spectrum increase [39]. For example, when the subjects perform the right hand motor imagery, it will correspondingly cause the left motion area to produce ERD, of which C3 is generally the most typical character. Same pheromone occurs with the left hand imagery. For one participant, we observed improvement after MI-BCI training by drawing ERSP graphs for the time-frequency analysis.

**Figure 2:**
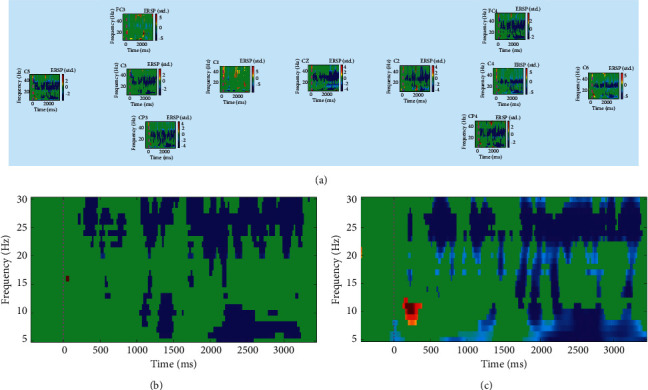
ERSP time-frequency measurements for session 1. (a) All channels, (b) C3, and (c) C4.

The all-channel ERSP diagram (Figure 2(a)) showed that in the early stage of rehabilitation training, in addition to FC3, C1, other channels presented two clusters of ERD: one centred at 5–10 Hz (ranging from about 2000 to 3000 ms), and the others centred at 20–30 Hz (ranging from about 500 to 3500 ms).

**Figure 3:**
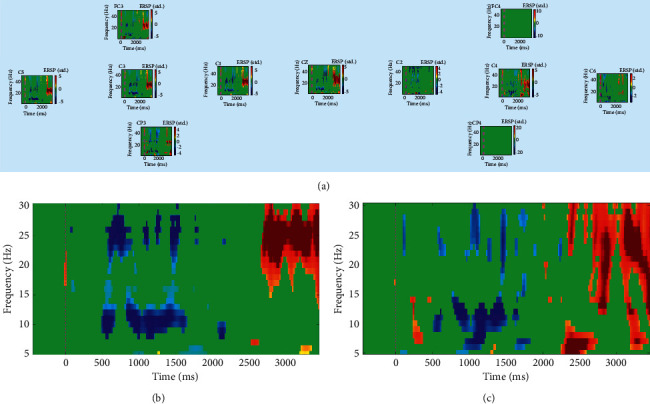
ERSP time-frequency measurements for session 11. (a) All channels, (b) C3, and (c) C4.

After 10 sessions of training (Figure 3), the ERSP of the left and right hemispheres differentiated. We could observe an alpha band of ERD appearing in the range of 10–15 Hz from 500 to 1500 ms in the left hemisphere. A beta band of ERS also appeared in the range of 20–30 Hz after 2500 ms. At the same time, only C4 in the right hemisphere showed a similar response.

**Figure 4:**
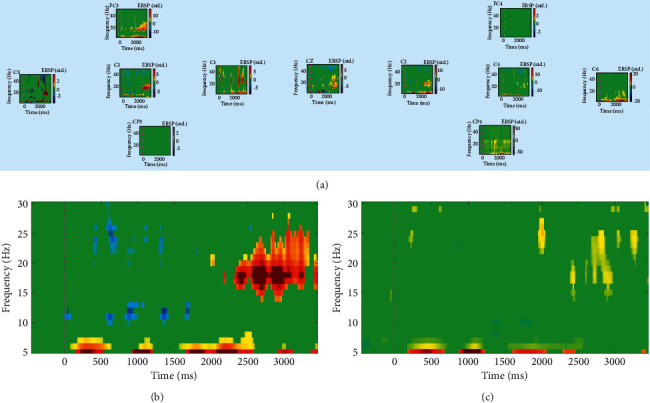
ERSP time-frequency measurements for session 19. (a) All channels, (b) C3, and (c) C4.

At session 19 (Figure 4), the participant's ERSP concentrated on a single channel. We could observe that C3 had an obvious ERS centred at 15–25 Hz in the range of 2500–3000 ms when compared with other channels.

### 5.2. Power Spectral Analysis

**Figure 5:**
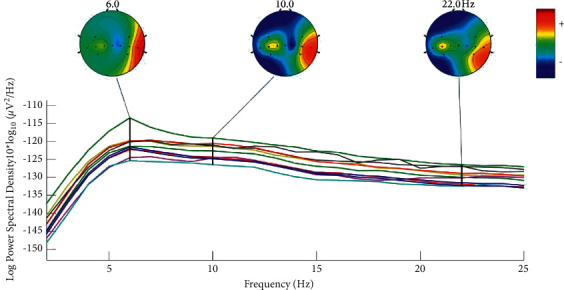
Power spectra analysis.

The PSD shows the power distribution of the EEG series in the frequency domain (Figure 5). Each coloured trace represents the spectrum of the activity of one data channel. The leftmost scalp map shows the scalp distribution of power at 6 Hz. The black vertical line corresponding to the scalp map shows all channels' log PSD and relative amplitude under a specific frequency band. Taking the 10th session of the same participants as an example, the PSD of all channels is the highest at 6 Hz, and the PSD of C6 is the highest among all channels.

## 6. Discussion

Stroke patients could benefit from rehabilitation therapy [40, 41]. However, the recovery of the upper limb is still a great challenge, especially for patients in chronic stage. In the current study, stroke patients received MI-based BCI training to see whether they could benefit more from this multimodal feedback training than the classic MI training. BCI technology has developed rapidly during the past few years. It does not require the involvement of peripheral nerves and muscles but allows the central nervous system to directly interact with the surrounding environment. Concerning its potential to promote brain remodelling and structural restructuring, it has been widely applied in neuro-rehabilitation together with exoskeleton robots, functional electric stimulation, and other end-effectors [15, 31, 42, 43]. Although, there was some heterogeneity among various studies, the effectiveness of BCI in motor recovery was worthy of affirmation [19, 26–28].

Mental task exercise in MI can be linked to the brain regions that control motor execution. Repeated participation of these motor regions during training might effectively promote brain plasticity and the obtained functional outcomes [44, 45]. Some studies have indicated that the patterns of neurons activated during mental tasks were similar to those generated during actual movement [46]. Thus, the motor patterns involved in actual functional activities might be enhanced during MI, which contributes to functional recovery; this theory has been validated by subsequent studies [47]. In terms of poststroke rehabilitation, task-oriented training was considered one of the most effective interventions [48]. In fact, though, we found that patients with severe upper limb paralysis may not be able to adapt to these options in clinical practice. Under these circumstances, MI is presented as an alternative of easy application that is economical and that has the possibility of being carried out by these patients, which we should take into account as a complement to clinical care.

In our previous study [34], we combined wrist CPM with MI-based BCI, finding that chronic stroke patients with severe upper limb motor impairment might improve paretic wrist ROM after the intervention. This might indicate that they could benefit from this therapy. However, it was a before and after study. To further investigate the effect of MI-based BCI in upper limb motor recovery, we compared MI-based BCI intervention with the classic MI to see their effect on the recovery of upper limb motor function in stroke patients. The following results were obtained from this study: MI-based BCI combined with multimodal feedback had beneficial effects on the recovery of severe upper limb impairments after stroke and had the potential to restore control of the paralysed wrist.

In the current study, the motor section of the FMA-UE score was used as the primary outcome. Before the intervention, there was no difference between the MI-BCI group and MI group. After the intervention, upper limb motor function improved more significantly in the MI-BCI group than in the MI group. However, when comparing the control of the paretic wrist, MI-BCI did not show a better efficacy. The following reasons might explain this: first, we found that five of the seven patients in the MI-BCI group were in the chronic stroke stage, while only one in the control group was in the chronic stage after stroke. Hence, disease duration might be a nonnegligible factor. In addition, MSS scores as secondary endpoints performed well in both groups. Further analysis revealed that the MSS scale could be subdivided into the shoulder elbow part and wrist hand part, and both groups showed excellent improvement in the shoulder elbow part, which produced effective functional movements (such as lifting the arm and touching the mouth in supine position). Previous study indicated that if stroke patients show no measurable grasp strength by four weeks and a prolonged flaccidity period, they may have poor prognosis of the hand [49]. Therefore, although the reemergence of wrist extension is relatively minor and cannot be immediately translated into functional activities, it has some meaning for patients with tiny residual hand function. Simultaneously, the reappearance of new movement increased the patient's confidence and mobilised their enthusiasm, which may help with long-term recovery.

There was no difference in the ARAT scores between the two groups. Both groups improved in the MSS score. However, further analysis indicated that the MSS scale could be subdivided into the shoulder elbow part and wrist hand part. Both groups showed excellent improvement in the shoulder and elbow, leading to the improvement for certain functional movements (such as lifting the arm and touching the mouth in supine position), whereas the score on the wrist hand part differed only in the control group.

The improvement of modified BI was not obvious between the two groups. There might be some possible explanations for this. First, the ultimate goal of rehabilitation is to help patients return to their families and society. During rehabilitation, patients have undergone standardised rehabilitation to increase their self-independence, regardless of their severity of motor impairment. Second, improvement in motor function did not directly equal improvement in ADL.

Our results also indicated that even in patients with central nervous system disease, MI ability could also be enhanced by multimodal feedback. MI could not be controlled well in the initial stage because of factors such as stroke and ERD appeared in almost all channels. This situation then improved after MI-based BCI training. The control level of the left hemisphere is relatively stronger than that of the right hemisphere, with ERD at the alpha band (5–10 Hz) and ERS at the beta band (20–30 Hz). In the later stage, the C3 channel had a clear ERS response at 20–30 Hz, indicating that this intervention could help improve the control of MI.

In the current study, we also investigated the security of MI-BCI. Conductive paste allergy, dizziness, and blurred vision might be common side effects. A questionnaire was surveyed, and none of the seven participants had the above adverse reactions.

The potential mechanism of BCI promoting motor function recovery is still being explored. However, it has been shown that BCI can change the neural network involved in motor recovery [4]. Studies have shown that the neural structures activated by MI are similar to those activated when certain movements are actually performed [50]. In addition, BCI is combined with an auditory, visual, or tactile system. All of these might reactivate and reorganize the brain structures and, through their feedback-based learning, lead to Hebbian-like plasticity-based cortical mechanisms. Generally, we believe that the ipsilateral limb is dominated by the contralateral brain. In fact, the ipsilateral motor cortex also contains important information about the state of the ipsilateral limb [51]. Therefore, even if the ipsilateral brain is severely damaged, the contralateral brain may also support the recovery of motor function through functional compensation. However, it is not unclear whether it is the recanalisation of the motor pathways on the affected side or the functional compensation on the healthy side that truly plays a role in the BCI system. According to the analysis of our EEG data, the brain remodelling of these patients may not be revealed by a single hypothesis, and the dual-mode equilibrium recovery may reflect the changes in brain remodelling after BCI training. In further studies, fMRI and diffusion tensor imaging (DTI) will be used to verify this hypothesis.

Therefore, we could conclude that MI-BCI could be used in stroke patients which might have some beneficial effect. There are still several limitations in the current study. First, it was only a preliminary study, and the sample size was not adequate enough to draw a conclusion. Second, improvement in upper limb motor function was still insufficient for functional use in daily life. This treatment may not substitute for the whole conventional rehabilitation therapy. Third, only the EEG signal of the participants in BCI group were collected and analysed, so the EEG patterns of the participants in the control group were not revealed and compared with those in BCI group. Forth, in the inclusion criteria, we did not standardize the motor function of the shoulder and elbow, which might affect the results of this study. Moreover, studies including motor-evoked potential (MEP) and DTI may further our understanding of the brain reorganisation mechanisms underlying this treatment.

## 7. Conclusion

Despite the current sample size being limited, the results suggest that stroke patients with severe restriction of residual upper limbs could gain a beneficial impact on motor function from MI-based BCI combined with multimodal feedback training. A potential in wrist extension movement was also observed. With further studies to provide stronger evidence, MI-based BCI combined with multimodal training might be a promising intervention for chronic stroke patients with severe upper limb impairment to further improve their motor function.

## Figures and Tables

**Table 1:** Demographic characteristics of the participants (*N* = 12).

Characteristics	MI-based BCI group (*n* = 7)	Classic MI group (*n* = 5)	*p* value
Sex			0.408
Male	4	4	
Female	3	1	
Age (yr)	44.9 ± 7.5	60.4 ± 16.8	0.053
Stroke type			0.558
Ischemic	3	3	
Hemorrhagic	4	2	
Affected side			0.091
Right	3	0	
Left	4	5	
Poststroke duration (mo)	7.9 ± 6.5	7.3 ± 4.5	0.868

Mean ± standard deviation was used for the different baseline characteristics. MI-based BCI = motor imagery-based brain-computer interface; classic MI = classic motor imagery.

**Table 2:** Patients' performance and treatment effects in all outcome measures and comparison of the treatment effects between the experimental and control groups after the interventions.

Parameters	MI-based BCI group (*n* = 7)		Classic MI group (*n* = 5)		*Sig*.^*a*^	*Sig*.^*b*^	*Sig*.^*c*^
	Baseline	After intervention	Baseline	After intervention			
FMA-UE	12.7(8.88)	15.4(10.11)	13.8(6.65)	20.6(12.67)	**0.006**	0.068	0.214
ARAT	3.29(5.79)	5.57(7.66)	6.60(12.29)	8.60(9.10)	0.089	0.058	0.375
MSS	15.9(8.97)	19.17(10.33)	18.60(10.98)	23.90(11.11)	**0.010**	**0.002**	0.112
BI	66.43(22.31)	69.00(11.93)	75.71(11.34)	72.00(6.71)	0.174	0.374	0.376
sEMG	2.55(1.83)	4.86(5.02)	2.14(1.95)	3.99(2.90)	0.305	0.130	0.866
	44.00(18.28)	43.90(16.43)	49.41(8.09)	57.20(12.24)	0.989	0.110	0.389

^a,b^Main effect of assessment (pre-post), within-group comparisons in MI-BCI and MI group, respectively. ^c^Interaction effect between treatment type (MI-BCI/MI) and assessment time (pre-post), between-groups comparisons, all significant *p* values are in bold. Mean (standard deviation) for all parameters. MI-based BCI = motor imagery-based brain-computer interface; classic MI = classic motor imagery; FMA-UE = Fugl-Meyer assessment-upper extremity; ARAT = action research arm test; MSS = motor status scale; BI = Barthel index; sEMG = the amplitude and cocontraction of the extensor carpi radialis on the surface electromyography. The bold values denote statistical significance.

## Data Availability

The original data could be processed by writing an email to Dr. Rongrong Lu (0356213@fudan.edu.cn).

## References

[1] Warlo C., Gijn J. van, Dennis M. (2008). *Stroke: Practical management*.

[2] Taub E., Uswatte G., King D. K., Morris D., Crago J. E., Chatterjee A. (2006). A placebo-controlled trial of constraint-induced movement therapy for upper extremity after stroke. *Stroke*.

[3] Van Delden A. E. Q., Peper C. E., Nienhuys K. N., Zijp N. I., Beek P. J., Kwakkel G. (2013). Unilateral versus bilateral upper limb training after stroke. *Stroke*.

[4] Ramos-Murguialday A., Broetz D., Rea M. (2013). Brain-machine interface in chronic stroke rehabilitation: a controlled study. *Annals of Neurology*.

[5] Schuster-Amft C., Eng K., Suica Z. (2018). Effect of a four-week virtual reality-based training versus conventional therapy on upper limb motor function after stroke: a multicenter parallel group randomized trial. *PLoS One*.

[6] Doyle S., Bennett S., Fasoli S. E. (2010). Interventions for sensory impairment in the upper limb after stroke[J]. *Cochrane Database of Systematic Reviews*.

[7] Tyson S. F., Hanley M., Chillala J., Selley A. B., Tallis R. C. (2008). Sensory loss in hospital-admitted people with stroke: characteristics, associated factors, and relationship with function. *Neurorehabilitation and Neural Repair*.

[8] Abbruzzese G., Berardelli A. (2003). Sensorimotor integration in movement disorders. *Movement Disorders*.

[9] Schabrun S., Hillier S. (2009). Evidence for the retraining of sensation after stroke: a systematic review. *Clinical Rehabilitation*.

[10] Ridding M. C., Brouwer B., Miles T. S., Pitcher J. B., Thompson P. D. (2000). Changes in muscle responses to stimulation of the motor cortex induced by peripheral nerve stimulation in human subjects. *Experimental Brain Research*.

[11] Edwards D. J., Dipietro L., Demirtas-Tatlidede A. (2014). Movement-generated afference paired with transcranial magnetic stimulation: an associative stimulation paradigm. *Journal of NeuroEngineering and Rehabilitation*.

[12] Kennedy P. R., Adams K. D. (2003). A decision tree for brain-computer interface devices. *IEEE Transactions on Neural Systems and Rehabilitation Engineering*.

[13] McConnell A., Moioli R., Brasil F. (2017). Robotic devices and brain-machine interfaces for hand rehabilitation post-stroke. *Journal of Rehabilitation Medicine*.

[14] Curado M. R., Cossio E. G., Broetz D. (2015). Residual upper arm motor function primes innervation of paretic forearm muscles in chronic stroke after brain-machine interface (BMI) training. *PLoS One*.

[15] Bundy D. T., Souders L., Baranyai K. (2017). Contralesional brain-computer interface control of a powered exoskeleton for motor recovery in chronic stroke survivors. *Stroke*.

[16] Prasad G., Herman P., Coyle D., McDonough S., Crosbie J. (2010). Applying a brain-computer interface to support motor imagery practice in people with stroke for upper limb recovery: a feasibility study. *Journal of NeuroEngineering and Rehabilitation*.

[17] Hong K.-S., Khan M. J. (2017). Hybrid brain-computer interface techniques for improved classification accuracy and increased number of commands: a review. *Frontiers in Neurorobotics*.

[18] Pfurtscheller G., Neuper C. (2006). Future prospects of ERD/ERS in the context of brain-computer interface (BCI) developments. *Progress in Brain Research*.

[19] Buch E., Weber C., Cohen L. G. (2008). Think to move: a neuromagnetic brain-computer interface (BCI) system for chronic stroke. *Stroke*.

[20] Ietswaart M., Johnston M., Dijkerman H. C. (2011). Mental practice with motor imagery in stroke recovery: randomized controlled trial of efficacy. *Brain*.

[21] Page S. J., Levine P., Leonard A. C. (2005). Effects of mental practice on affected limb use and function in chronic stroke. *Archives of Physical Medicine and Rehabilitation*.

[22] Sirigu A., Cohen L., Duhamel J. R. (1995). Congruent unilateral impairments for real and imagined hand movements. *NeuroReport*.

[23] Dijkerman H. C., Ietswaart M., Johnston M., MacWalter R. S. (2004). Does motor imagery training improve hand function in chronic stroke patients? A pilot study. *Clinical Rehabilitation*.

[24] Guttman A., Burstin A., Brown R., Bril S., Dickstein R. (2012). Motor imagery practice for improving sit to stand and reaching to grasp in individuals with poststroke hemiparesis. *Topics in Stroke Rehabilitation*.

[25] Kim S.-S., Lee B.-H. (2015). Motor imagery training improves upper extremity performance in stroke patients. *Journal of Physical Therapy Science*.

[26] Ang K. K., Guan C., Phua K. S. (2012). Transcranial direct current stimulation and EEG-based motor imagery BCI for upper limb stroke rehabilitation. *Annual International Conference of the IEEE*.

[27] Pichiorri F., Morone G., Petti M. (2015). Brain-computer interface boosts motor imagery practice during stroke recovery. *Annals of Neurology*.

[28] Mattia D., Pichiorri F., Colamarino E. (2020). The Promotoer, a brain-computer interface-assisted intervention to promote upper limb functional motor recovery after stroke: a study protocol for a randomized controlled trial to test early and long-term efficacy and to identify determinants of response. *BMC Neurology*.

[29] Laver K. E., Lange B., George S. (2017). Virtual reality for stroke rehabilitation[J]. *Cochrane Database of Systematic Reviews*.

[30] Vourvopoulos A., Bermúdez i Badia S. (2016). Motor priming in virtual reality can augment motor-imagery training efficacy in restorative brain-computer interaction: a within-subject analysis. *Journal of NeuroEngineering and Rehabilitation*.

[31] Frolov A. A., Mokienko O., Lyukmanov R. (2017). Post-stroke rehabilitation training with a motor-imagery-based brain-computer interface (BCI)-Controlled hand exoskeleton: a randomized controlled multicenter trial. *Frontiers in Neuroscience*.

[32] Kim T., Kim S., Lee B. (2016). Effects of action observational training plus brain-computer interface-based functional electrical stimulation on paretic arm motor recovery in patient with stroke: a randomized controlled trial. *Occupational Therapy International*.

[33] Ramos-Murguialday A., Curado M. R., Broetz D. (2019). Brain-Machine interface in chronic stroke: randomized trial long-term follow-up. *Neurorehabilitation and Neural Repair*.

[34] Lu R.-R., Zheng M.-X., Li J. (2020). Motor imagery based brain-computer interface control of continuous passive motion for wrist extension recovery in chronic stroke patients. *Neuroscience Letters*.

[35] Malouin F., Richards C. L., Jackson P. L., Lafleur M. F., Durand A., Doyon J. (2007). The kinesthetic and visual imagery questionnaire (KVIQ) for assessing motor imagery in persons with physical disabilities: a reliability and construct validity study. *Journal of Neurologic Physical Therapy*.

[36] https://sccn.ucsd.edu/wiki/Makoto’s_preprocessing_pipeline.

[37] Page S. J., Levine P., Sisto S. A., Johnston M. V. (2001). Mental practice combined with physical practice for upper-limb motor deficit in subacute stroke. *Physical Therapy*.

[38] Page S. J., Levine P., Sisto S., Johnston M. V. (2001). A randomized efficacy and feasibility study of imagery in acute stroke. *Clinical Rehabilitation*.

[39] Cebolla A. M., Petieau M., Cevallos C., Leroy A., Dan B., Cheron G. (2015). Long-lasting cortical reorganization as the result of motor imagery of throwing a ball in a virtual tennis court. *Frontiers in Psychology*.

[40] Hebert D., Lindsay M. P., McIntyre A. (2016). Canadian stroke best practice recommendations: stroke rehabilitation practice guidelines, update 2015. *International Journal of Stroke*.

[41] Lin K.-C., Chung H.-Y., Wu C.-Y. (2010). Constraint-Induced therapy versus control intervention in patients with stroke. *American Journal of Physical Medicine & Rehabilitation*.

[42] Jovanovic L. I., Kapadia N., Lo L., Zivanovic V., Popovic M. R., Marquez-Chin C. (2020). Restoration of upper limb function after chronic severe hemiplegia. *American Journal of Physical Medicine & Rehabilitation*.

[43] Mukaino M., Ono T., Shindo K. (2014). Efficacy of brain-computer interface-driven neuromuscular electrical stimulation for chronic paresis after stroke. *Journal of Rehabilitation Medicine*.

[44] Cicinelli P., Marconi B., Zaccagnini M., Pasqualetti P., Filippi M. M., Rossini P. M. (2006). Imagery-induced cortical excitability changes in stroke: a transcranial magnetic stimulation study. *Cerebral Cortex*.

[45] Sharma N., Baron J.-C. (2013). Does motor imagery share neural networks with executed movement: a multivariate fMRI analysis. *Frontiers in Human Neuroscience*.

[46] Hétu S., Grégoire M., Saimpont A. (2013). The neural network of motor imagery: an ALE meta-analysis. *Neuroscience & Biobehavioral Reviews*.

[47] Fernandez-Gomez E., Sanchez-Cabeza A. (2018). Motor imagery: a systematic review of its effectiveness in the rehabilitation of the upper limb following a stroke[J]. *Revue Neurologique*.

[48] Van Peppen R. P., Kwakkel G., Wood-Dauphinee S., Hendriks H. J., Van der Wees P. J., Dekker J. (2004). The impact of physical therapy on functional outcomes after stroke: what’s the evidence?. *Clinical Rehabilitation*.

[49] Twitchell T. E. (1951). The restoration of motor function following hemiplegia in man. *Brain*.

[50] Jeannerod M., Decety J. (1995). Mental motor imagery: a window into the representational stages of action. *Current Opinion in Neurobiology*.

[51] Ganguly K., Secundo L., Ranade G. (2009). Cortical representation of ipsilateral arm movements in monkey and man. *Journal of Neuroscience*.

